# Potential Parasitoids for Biocontrol of the Ber Fruit Fly, *Carpomya vesuviana* Costa (Diptera: Tephritidae)

**DOI:** 10.3390/life14010050

**Published:** 2023-12-28

**Authors:** Alieh Amini, Hossein Lotfalizadeh, Francisco Javier Peris-Felipo, Jean-Yves Rasplus

**Affiliations:** 1 Department of Plant Protection, Ferdowsi University of Mashhad, Mashhad P.O. Box 91779-48974, Iran; amini.alieh@yahoo.com; 2 Insect Taxonomy Research Department, Iranian Research Institute of Plant Protection (IRIPP), AREEO, Tehran P.O. Box 19395-1454, Iran; 3 Independent Researcher, Bleichestrasse 15, 4058 Basel, Switzerland; peris.felipo@gmail.com; 4 CBGP, University Montpellier, CIRAD, INRA, IRD, Montpellier SupAgro, 34060 Montpellier, France; jean-yves.rasplus@inrae.fr

**Keywords:** parasitoids, Eurytomidae, Pteromalidae, *Aximopsis*, *Eurytoma*, jujube, medical plant

## Abstract

The ber fruit fly (BFF), *Carpomya vesuviana* Costa, 1854 (Diptera: Tephritidae), is an important key pest of the jujube, *Ziziphus jujuba* Miller. The main control measures against this pest are to use chemical control, but the first survey for its natural enemies was performed in Iran. Here, we report eight species of parasitic wasps of the BFF from five different families. The family Eurytomidae with three species, the families Pteromalidae and Mutillidae with two species each, and the families Braconidae and Diapriidae with one species each are associated with different immature stages of the BFF, of which *Eurytoma pineticola* Zerova (Eurytomidae) and *Cyrtoptyx lichtensteini* (Masi) (Pteromalidae) were the most abundant parasitoid species. *Fopius carpomyiae* (Silvestri,) was not reared on BFF on the jujube during this survey, but it was reported on *Ziziphus spina-christi* (L.) with a high parasitism rate. Therefore, it seems to be the most important parasitoid of BFF in Iran. The parasitoid community of BFF in Khorasan, Eastern Iran, is reviewed, and an identification key to these species is proposed.

## 1. Introduction

The genus *Ziziphus* (Rhamnaceae), with about 135 species, is mostly distributed in the tropical and subtropical regions of Asia and America [1] and a fewer number of species in the Pacific Islands and Australia [2,3].

Some species in the genus are of tremendous economic and medicinal importance. The fruits of *Ziziphus jujuba* Miller, known as jujube or Chinese date, are employed medicinally to treat human anxiety and insomnia [4]. The fruits are also used to alleviate stress, as an appetite stimulant, as a digestive aid, and for antiarrhythmic and contraceptive medication [5,6,7].

The majority of the Iranian territory has a Mediterranean climate, but Southern and Eastern Iran have sub-tropical and semi-arid climates [8]. Thus, the country has a favorable climate for tropical fruit crops, such as jujube. This plant is associated with more than 20 species of arthropod pests [9,10] including caterpillars, beetles, and mites [11,12,13]. Among them, the ber fruit fly (BFF), *Carpomya vesuviana* Costa, 1854 (Diptera: Tephritidae), is an economically important pest with a high population level and the highest level of infestation of jujube fruits [14,15]. As a key pest of *Ziziphus*, the fly can cause serious damage and yield loss of up to 80–100% fruit infestation [15,16].

BFF is widely distributed in southern European countries and Asia [17]. In Iran, this species also attacks a few other cultivated *Ziziphus* species [18]. BFF is also one of the most destructive pests for *Ziziphus* in adjacent countries (China, India, Middle East, and Pakistan) [10,12,14,15,18,19]. The morphology of the larva and the biological characteristics of BFF were described by Kandybina [20] and Hu et al. [21]. Jena et al. [22] mentioned the absence of a successful record of the BFF natural enemies in India.

Until now, only a few parasitoids have been reported. Among them are three braconid species [15,23], of which *Fopius carpomyiae* (Silvestri, 1916) is the most studied parasitoid [15,17,19,24,25]. This species was also reported as a parasitoid of BFF infesting *Ziziphus spina-christi* (L.) Willd in southern Iran [26]. Narayanan and Chawal [27] reported five braconids and an eulophid species from India. To date, no chalcidoid parasitoids have been listed from BFF in the Universal Chalcidoidea Database [28], but *Eupelmus urozonus* Dalman, 1820 was reported on *Carpomya incompleta* (Becker, 1903), a closely related species [29]. Amini et al. [30] reported two larval and pupal parasitoids of the BFF, while in a second report, they added two mutillid species reared from puparia of BFF [31]. A recent sampling of BFF in eastern Iran led to the discovery of several parasitoid species presented in this paper that can be potential biocontrol agents of BFF. Furthermore, we present the first review of known parasitoids associated with BFF infesting jujube in Iran and provide new information based on a dense survey of fruits infested by BFF.

## 2. Materials and Methods

BFF, *Carpomya vesuviana* larvae and puparia were collected from *Ziziphus jujuba* and soil and litter during January to October of 2012–2013 and 2017. Samplings were carried out from eight different localities in South Khorasan province, Eastern Iran. The collection of infested fruits was made from sites not treated with pesticides in Birjand County (Birjand, Chahkand, Mood, Noghab, Rakat, Razg, Zini) and Gaen County (Shahrakht). The ripe and green jujube fruits were placed in paper bags in coolers and transported to our laboratory in the Entomology laboratory of the Department of Plant Protection, Ferdowsi University, Mashhad. These samples were kept separately per collecting batch to rear their parasitoids. About 2150 (≈17 kg) fruits of *Z. jujuba* were dissected, and infested fruits were individually placed in plastic rearing jars (r = 2.5 cm, h = 10 cm) and sprayed with water on a weekly basis to maintain moisture. BFF’s puparia were collected from soil and litter under the jujube trees, and rearing was performed in laboratory conditions (24 ± 3 °C, 70%RH) to obtain possible parasitoids. Emerging flies and parasitoids were collected and preserved in 70% ethanol. The parasitism rate of the obtained wasps was estimated by considering the number of infested fruits in each batch and the number of emerged parasitoids. Obtained data were analyzed using SPSS 26 statistical software and mean parasitism rates comparisons were performed using Duncan’s test.

Identification of parasitic wasps was made using available keys and descriptions [32,33,34,35,36,37,38,39,40,41,42,43,44,45]. All morphological terminology follows the Hymenoptera Anatomy Ontology (http://portal.hymao.org/, accessed on 10 January 2023). Images were taken with a Keyence digital microscope (VHX-5000). The final illustrations were edited and processed for contrast and brightness using Adobe Photoshop^®^ CS6 software. Voucher specimens were deposited in the HMIM and NHMUK.

## 3. Results

**Figure 1:**
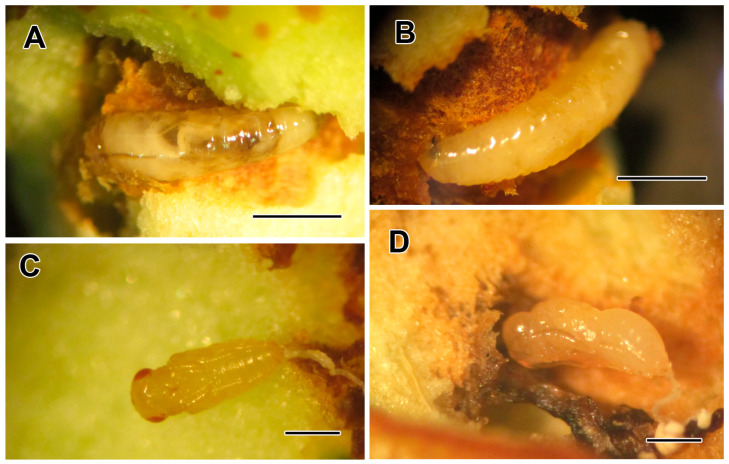
(**A**) Early third larval instar and (**B**) late third larval instar of BFF, *Carpomya vesuviana* in damaged fruit of *Ziziphus jujuba* fruit; (**C**,**D**) pupae of *C. vesuviana* parasitoids from the family Pteromalidae (scale bar, 1 mm).

Our large sampling and previously collected specimens revealed the presence of nine hymenopterous parasitoid species belonging to five different families (Braconidae, Diapriidae, Eurytomidae, Pteromalidae and Mutillidae) that mostly attack the larval and pupal forms of BFF (Figure 1A,B) (Table 1). A braconid species was reported from southern Iran as parasitoid of BFF; therefore, it was included in Table 1, while it was not obtained in our rearing from eastern Iran. Bushehr has a hot and humid climate with a mean temperature of 25 °C (up to 50 °C in summer) and a total annual rainfall of 170 mm, which is similar to the Indian climate, the origin country of *F. carpomyiae*. South Khorasan in eastern Iran has a desert and dry climate with a mean annual temperature of 33 °C in summer and 14 °C in winter, with a total annual rainfall of 134 mm, which seems inappropriate for this parasitoid.

### 3.1. Biological Data

Parasitoids of BFF are mainly active during June–July when the host is in the larval–puparial stages and the temperature is about 30 °C, which is the favorable temperature for pupal development and adult emergence [22]. The total parasitism rate of the presented parasitoids is about 5.50%, considering widely applied chemical pesticides against different groups of pests in the field condition can be a promising rate.

**Figure 2:**
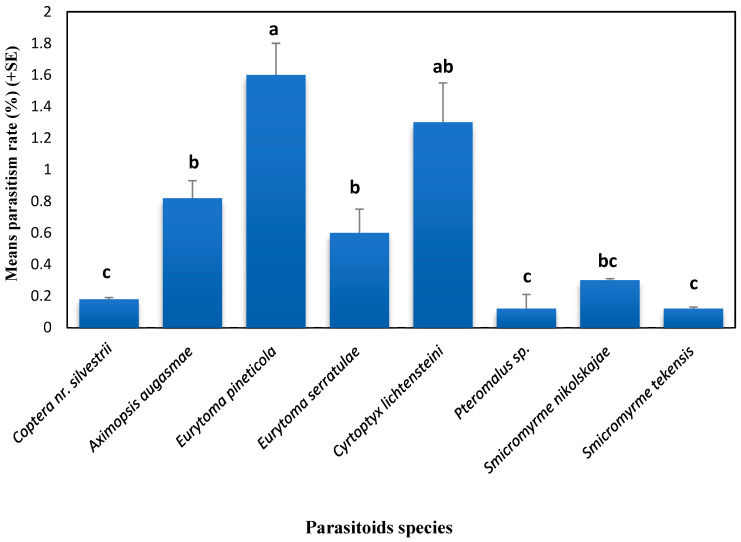
Parasitism rates of hymenopterous parasitoids of BFF, *Carpomya vesuviana* (Diptera: Tephritidae) infesting jujube fruit, *Ziziphus jujuba* in Khorasan province, Eastern Iran (bars topped by similar letters are not significantly different (Duncan’s test, *a* = 1%)).

The most abundant parasitoids reared were *Eurytoma pineticola* and *Cyrtoptyx lichtensteini*, which are larval ectoparasitoids of *C. vesuviana* with 1.60% and 1.30% parasitism rates, respectively (Figure 2).

### 3.2. Taxonomic Classification

***Fopius carpomyiae* (Silvestri, 1916)** (Hymenoptera: Braconidae)

**Diagnosis**. *Fopius carpomyiae* has a pale coloration body; frons densely covered with large punctures; second metasomal tergite striate, with a relatively short ovipositor, and the ovipositor tip is not narrowed apically. The species is closely related to *Fopius arisanus* (Sonan, 1932) and *F. vandenboschi* (Fullaway, 1952) but has a shorter ovipositor. Wharton [46] provided a table of morphological features that facilitate the comparison of known species of *Fopius*.

**Remark**. Farrar and Chou [26] believe this Oriental species [47,48] has been recently introduced to Iran and stablished in Bushehr’s orchards, southern Iran. We did not sample it from our rearing, but the species was reported from southern Iran as an egg–larval endoparasitoid of BFF on *Ziziphus spina-christi* by Farrar and Chou [26]. The percentage parasitism of this wasp was estimated to be 24% in Tangestan, Bushehr province [24].

*Galesus* nr. *silvestrii* (Kieffer, 1913) (Hymenoptera: Diapriidae)

**Material examined**: 1♀, Iran: South Khorasan province, Mood (59°31′23″ E, 32°42′31″ N), 1851 m, 20.vii.2012, A. Amini, leg. 2♀♀, Birjand (32°51′59″ N, 59°13′55″ E), 1465 m, 11.vii.2012, A. Amini, leg. 1♂, Birjand, Chahkand (32°50′49″ N, 59°09′20″ E), 1551 m, 21.vii.2012, A. Amini leg.

**Remark**. This species was reared from puparia of BFF. The species of the genus *Galesus* are well-known parasitoids of fruit flies. Our reared specimens were compared by David Notton, a Diapriidae specialist, with identified specimens in NHMUK [31] and related publications (such as 43). He found it to be similar to *Galesus silvestrii* in body color and sculpturing on the sclerites. Considering that these parasitic wasps received little attention in the Palaearctic region and that there is no updated reliable key for species discrimination, its identification remains uncertain.

*Galesus* as a genus native to North Africa is very easy to recognize, although it was previously included in the genus *Psilus*. In Palaearctic keys; for example, Nixon [40] and Kozlov [43]; it keys out as *Psilus*. Muesebeck [39] separated *Coptera* with, most obviously, the head shape with transverse carina in front of the occipital carina, longitudinally folded wings usually with an apical notch, and very short apical gastral segments.

**Figure 3:**
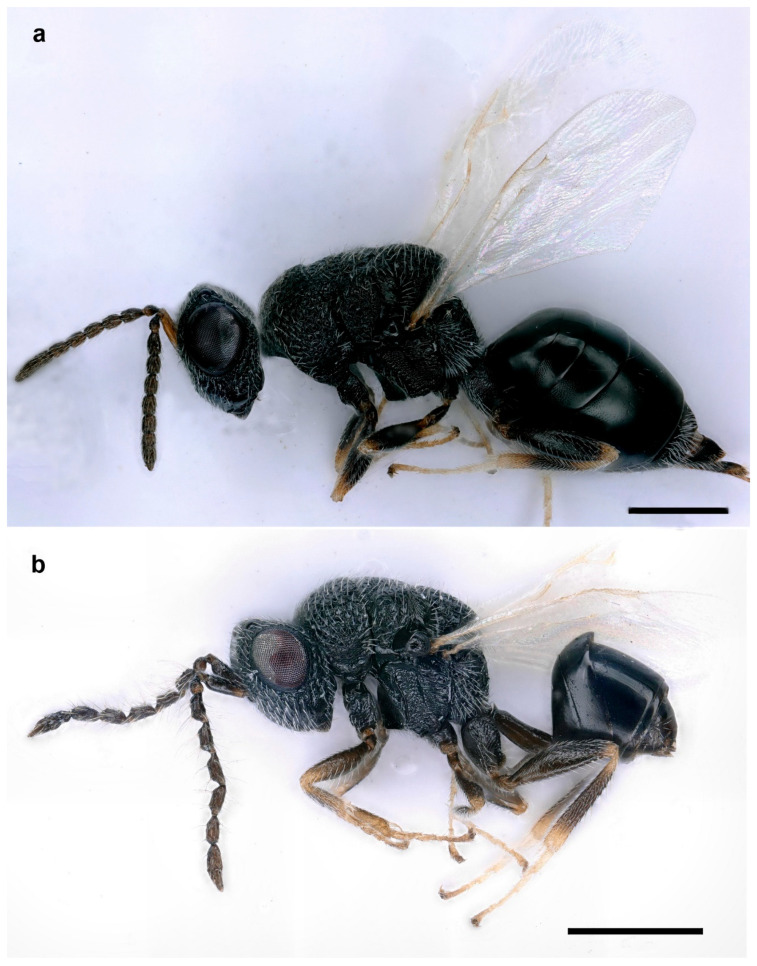
*Aximopsis augasmae*: (**a**) female, lateral habitus; (**b**) male, lateral habitus (scale bar, 500 µm).

**Figure 4:**
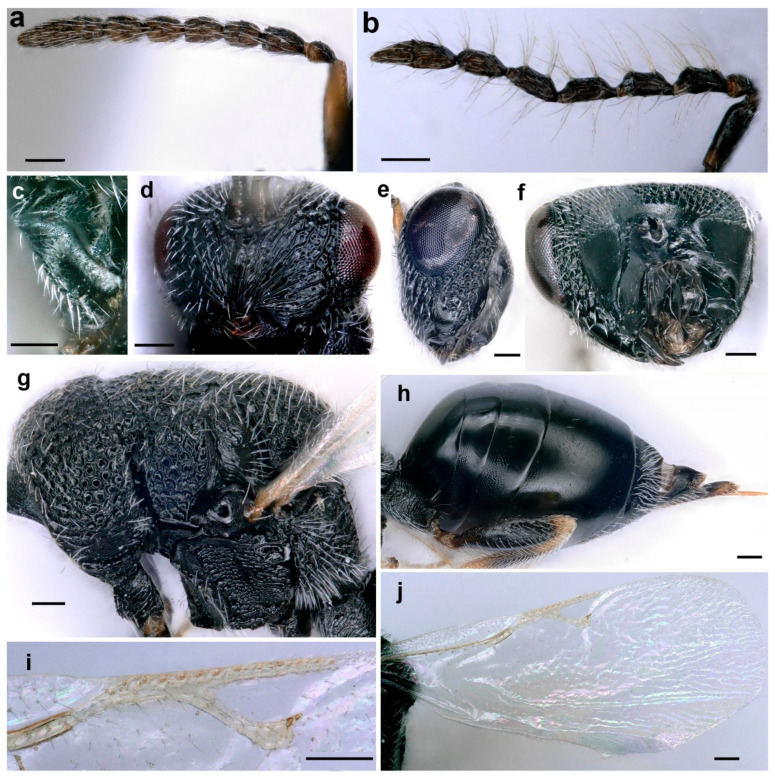
*Aximopsis augasmae*: (**a**) female antenna; (**b**) male antenna; (**c**) fore coxa; (**d**) head, fronto–ventral view; (**e**) head, lateral view; (**f**) head, posterior view; (**g**) mesosoma, lateral view; (**h**) metasoma, lateral view; (**i**) venation; (**j**) fore wing (scale bar, 100 µm).

*Aximopsis augasmae* (Zerova, 1977) (Hymenoptera: Eurytomidae) (Figure 3 and Figure 4)

**Material examined:** 15♀♀, 3♂♂, Iran: South Khorasan province, Birjand (32°51′59″ N, 59°13′55″ E), 1465 m, vii.2017, A. Amini, leg.

**Diagnosis**. The most important characters to distinguish *A. augasmae* from closely related species were discussed by Zerova and Khodzhevanishvili [38], Zerova and Seryogina [45] and Lotfalizadeh and Hosseini [49].

This species is characterized by the following characters: body generally black, with some parts brown (legs and antenna partly) (Figure 3a,b). Gena distinctly carinate posteriorly, postgenal lamina present and raised, joining genal carina ventrally (Figure 4e,f). Clypeus not delimited dorsally, strigose, truncate on ventral margin (Figure 4d). All funiculars longer than broad in females (Figure 4a); often asymmetric, strongly tapering apically and with whorls of long hairs in males (Figure 4b). Mesepimeron strigose, with a horizontal ventral shelf in front of mesocoxal foramina (Figure 4g). Mesodiscrimen with projecting triangular tooth in front of mesocoxal foramina (Figure 4g), epicnemium completely delimited. Postmarginal vein distinctly longer than stigmal and marginal veins (1.2 and 1.8 times, respectively) (Figure 4i,j). Procoxa with an oblique carina on the frontal side, delimiting, ventrally, a depression (Figure 4c). Metasoma as long as mesosoma and head together (Figure 3a), with a short petiole, shorter than width; horizontal ovipositor sheaths (Figure 4h).

**Remark**. BFF infesting *Z. jujuba* represents a new host for *A. augasmae* that develops as a solitary endoparasitoid of the larvae. This species is distributed in the eastern part of the West Palaearctic [28] and has been previously reared on *Augasma atraphaxidellum* Kuznetz (Lepidoptera: Coleophoridae) on *Atraphaxis spinosa* L. (Polygonaceae) and other moth species on *Zygophillum* [45], as well as *Etiella zinckenella* (Treitschke) (Lepidoptera: Pyralidae) on *Sophora alopecuroides* L. (Leguminosae) [49].

**Figure 5:**
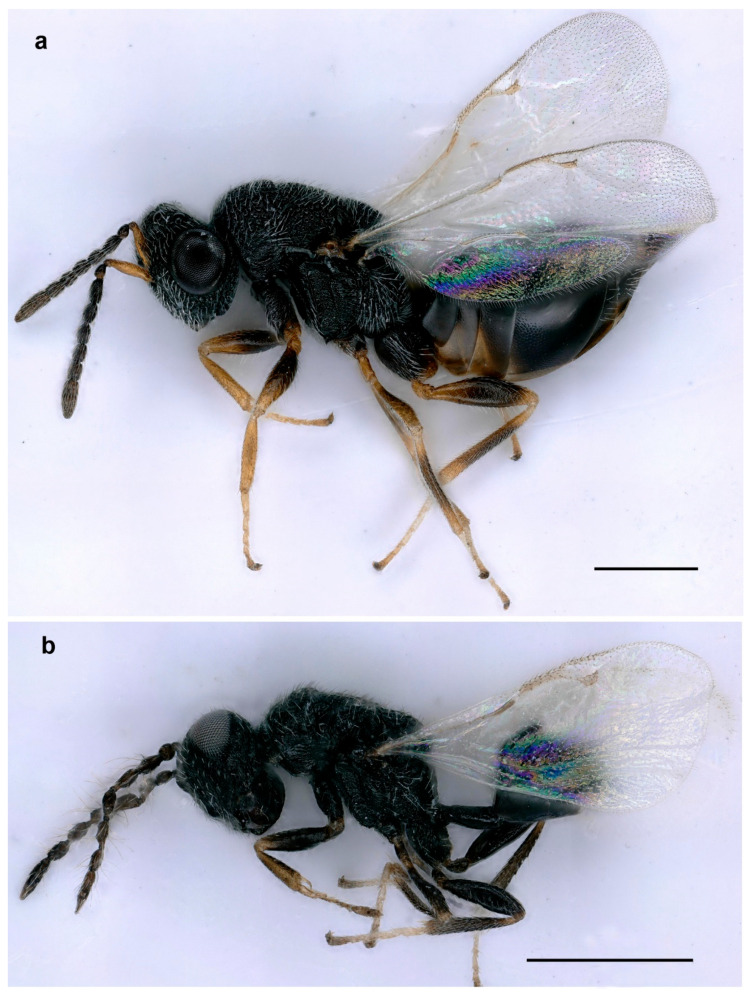
*Eurytoma pineticola*, lateral habitus: (**a**) female; (**b**) male (scale bar, 500 µm).

**Figure 6:**
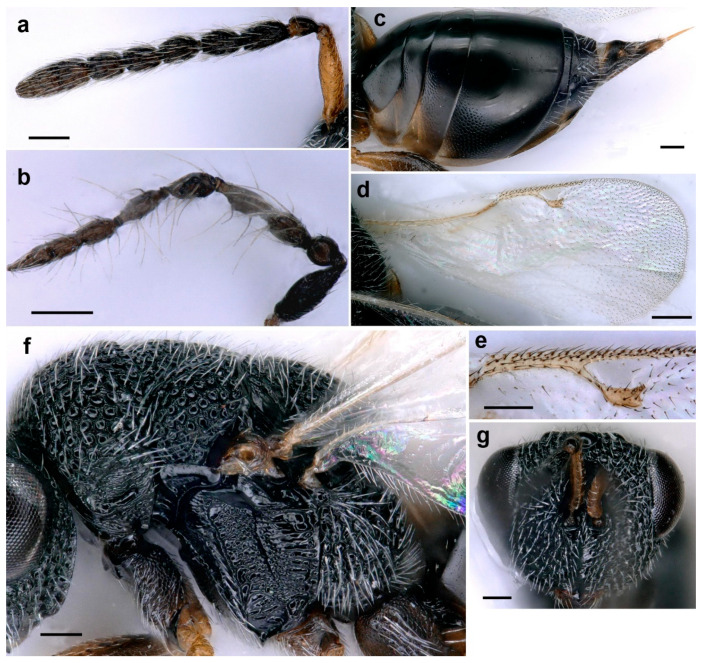
*Eurytoma pineticola*: (**a**) female antenna; (**b**) male antenna; (**c**) metasoma, lateral view; (**d**) fore wing; (**e**) venation; (**f**) mesosoma, lateral view; (**g**) head, frontal view (scale bar, 100 µm).

*Eurytoma pineticola* Zerova, 1981 (Hymenoptera: Eurytomidae) (Figure 5 and Figure 6).

**Material examined:** 25♀♀, 12♂♂, Iran: South Khorasan province, Birjand (32°51′59″ N, 59°13′55″ E), 1465 m, vi.2017, A. Amini, leg.

**Diagnosis**. *Eurytoma pineticola* is characterized by a strigose supraclypal area (Figure 6g), gena as long as eye height; funiculars in females distinctly longer than wide (Figure 6a), antennae of males with five funiculars and two clavomeres (Figure 6b); adscrobal carina of mesopleuron not elbowed and exhibiting a unique tooth in front of mesocoxa, mesepisternal shelf not developed (Figure 6f); marginal vein 1.5 times as long as stigmal vein (Figure 6e); metasoma as long as mesosoma (Figure 5a), slightly compressed laterally and slightly elevated at apex (Figure 6c); Gt4 is the largest tergite.

*Eurytoma pineticola* is closely related to *Eurytoma serratulae* (Fabricius) and can be separated by the characters mentioned in the key.

**Remark**. This species was reared for the first time on larvae of BFF infesting *Z. jujuba*. However, it was already reported from non-frugivorous tephritid such as *Paratephritis transitoria* (Rohdendorf) in the flower head of *Parasenecio hastatus* (L.) H.Koyama (Asteraceae) [41].

**Figure 7:**
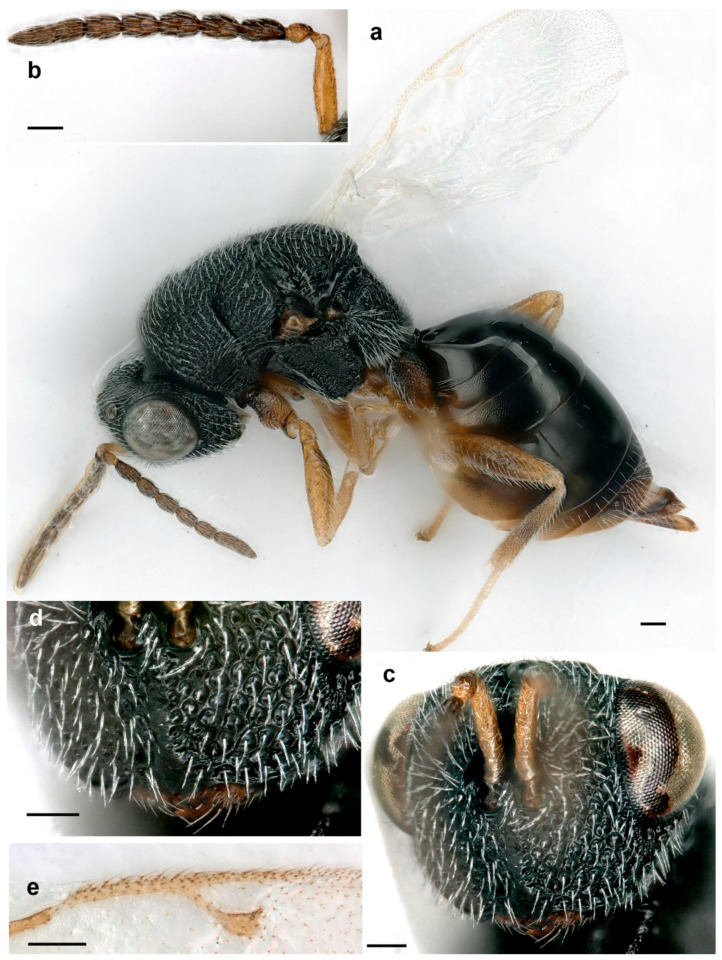
*Eurytoma serratulae*, female: (**a**) lateral habitus; (**b**) antennae; (**c**) head, frontal view; (**d**) lower face and clypeus; (**e**) fore wing venation (scale bar, 100 µm).

**Figure 8:**
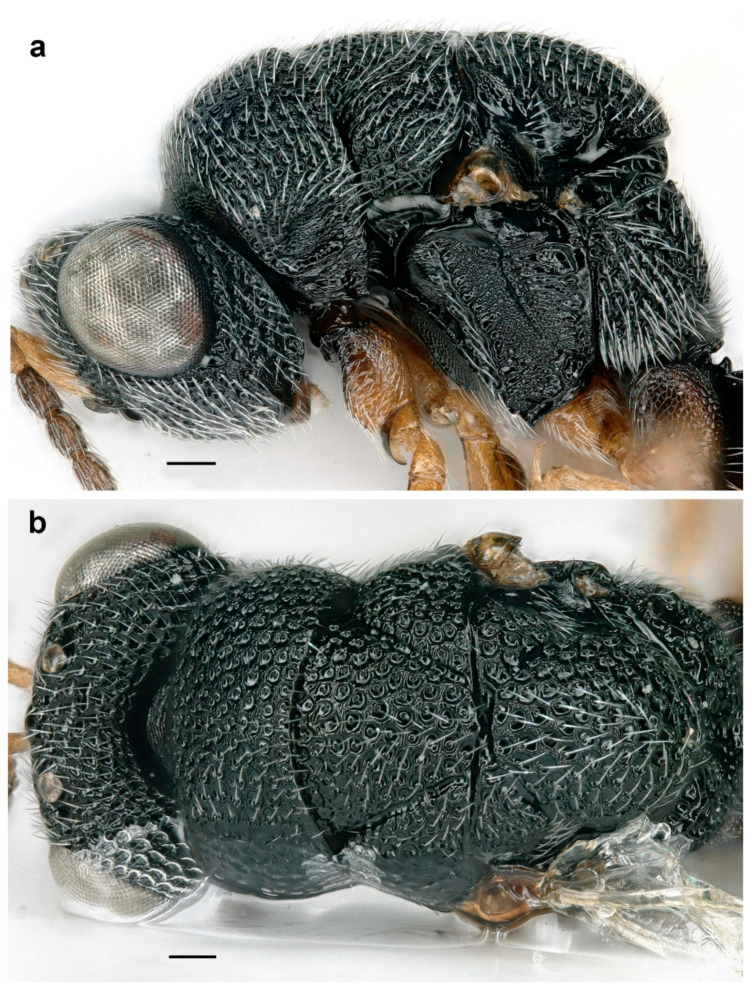
*Eurytoma serratulae*, female, head and mesosoma: (**a**) lateral view; (**b**) dorsal view (scale bar, 100 µm).

**Figure 9:**
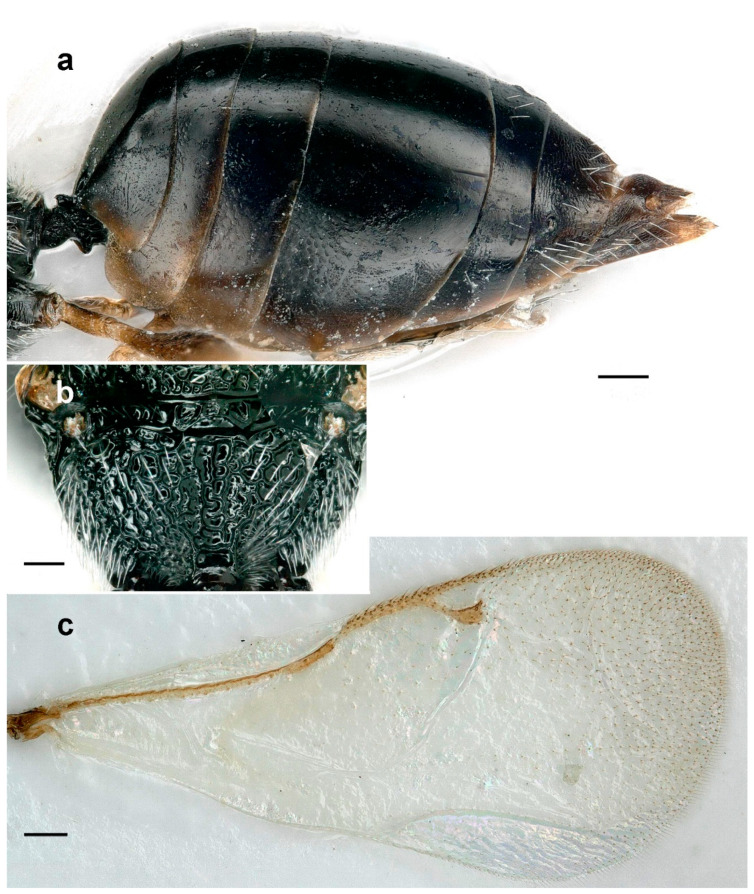
*Eurytoma serratulae*, female: (**a**) metasoma, lateral view; (**b**) propodeum, dorsal view; (**c**) fore wing (scale bar, 100 µm).

*Eurytoma serratulae* (Fabricius, 1798) (Hymenoptera: Eurytomidae) (Figure 7, Figure 8 and Figure 9).

**Material examined:** 14♀♀, Iran: South Khorasan province, Birjand (32°51′59″ N, 59°13′55″ E), 1465 m, vii.2017, A. Amini, leg.

**Diagnosis**. *Eurytoma serratulae* is characterized by its punctured supraclypeal area (Figure 7c,d), clypeal margin slightly emarginate (Figure 7d); gena conspicuously carinate posteriorly, postgena with ventral depression and posterior margin of gena forming a blunt angle and emarginate lateral to edge of oral fossa; postgenal lamina present, ventrally raised, in lateral view appearing as a tooth. In females, funiculars longer than wide (Figure 7a), with two clavomeres that are nearly always fused; notauli complete and not obliterated (Figure 8b), axillar groves with a pit at mid-length, obliterated by sculpture anteriorly, their bottom thus not clearly visible; epicnemium not entirely delimited, adscrobal carina of mesopleuron not elbowed, forming a single tooth in front of mesocoxa (Figure 8a); propodeum with a median furrow, not impressed and situated in a broadly concave surface (Figure 9b); ovipositor sheaths relatively horizontal (Figure 9a).

This species is close to *Eurytoma compressa* (Fabricius, 1794), and females can be separated by the shape of metasoma. While the metasoma of *E. serratulae* is elongated, slightly flattened laterally and relatively less raised dorsally with a shorter ovipositor (Figure 8a), the metasoma of *E. compressa* females in the lateral view is round, strongly flattened, with a vertically raised Gt7 and a long ovipositor.

**Remark**. *Eurytoma serratulae* was reared for the first time from larvae of BFF. This species was previously reported as a parasitoid of other fruit flies, *Myopites longirostris* (Loew, 1846) (under *M. frauenfeldi* (Schiner, 1868)) [50], *Tephritis stictica* Loew, 1862 [50], *Urophora cardui* (L., 1758) [51] and an unknown species of *Urophora* sp. [34]. *Eurytoma pineticola* belongs to the *serratulae* species group that occurs in the Holarctic region and parasitizes gall-inducing Cynipidae (Hymenoptera), Tephritidae (Diptera) and Curculionidae (Coleoptera) [34,52]. Claridge [35] suggested that *E. serratulae* only parasitized gall-maker tephritids in stems of Asteraceae, but our finding demonstrated that the species can also parasitize larvae of fruit flies on Rhamnaceae.

*Cyrtoptyx lichtensteini* (Masi, 1921) (Hymenoptera: Pteromalidae)

**Material examined:** 24♀♀, 6♂♂, Iran: South Khorasan province, Birjand (32°51′59″ N, 59°13′55″ E), 1465 m, vii.2017, A. Amini, leg.

**Diagnosis**. The most important characteristics of *C. lichtensteini* were summarized by Lotfalizadeh and Hosseini [49] and Mete and Lotfalizadeh [53]. See Figure 3 in Mete and Lotfalizadeh [53] for the morphological details of this species.

The main morphological characters of the species are as follows: body black with bluish-green reflections on mesosoma, metasoma with reddish-blue reflections laterally and Gt1 with greenish reflections dorsally. Tibiae and tarsi are mainly whitish. Antennal formula 1,1,3,5,3, scape slightly exceeding the anterior ocellus, all funiculars longer than wide, Fu1 about twice as long as wide, clava 2.5 times as long as wide. Marginal vein longer than postmarginal and stigmal veins; relative measurements: marginal vein: 13; stigmal vein: 10; postmarginal vein: 12. Metasoma is about 2.2 times as long as broad and about 1.25 times as long as mesosoma.

**Remark**. This species is an ectoparasitoid of the larval stage, and it was reported on Coleoptera, Diptera, Hymenoptera and Lepidoptera [49,53] and widely distributed in the Palaearctic (from Europe and North Africa to China) and Nearctic regions [28].

**Figure 10:**
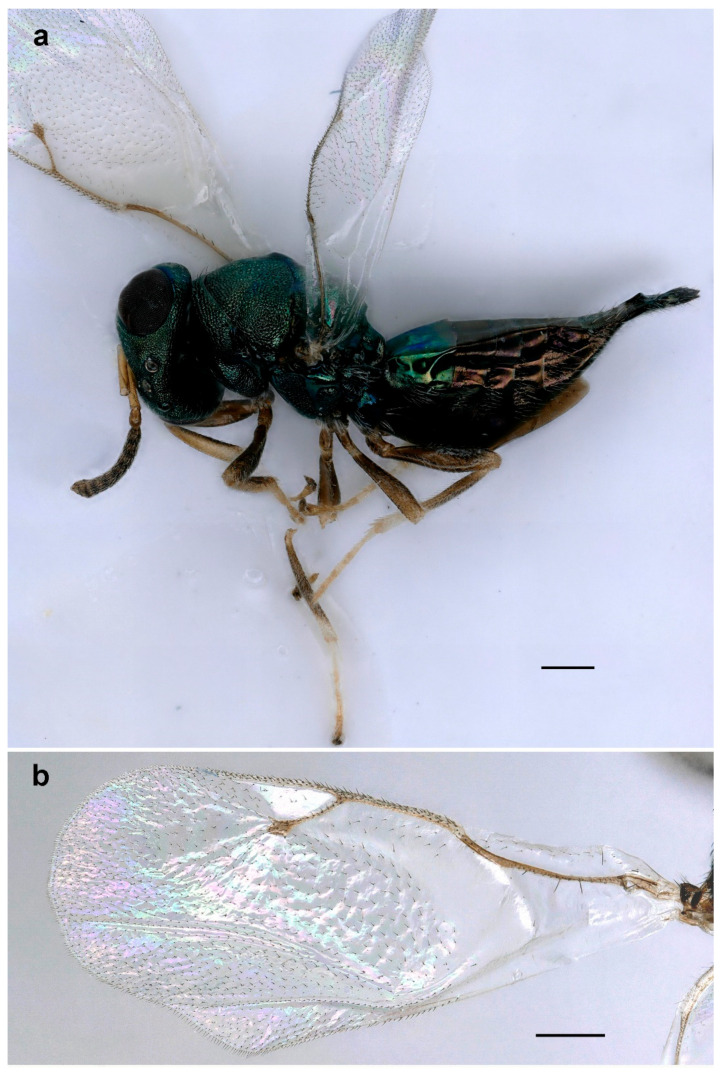
*Pteromalus* sp., female: (**a**) female, lateral habitus; (**b**) fore wing (scale bar, 200 µm).

**Figure 11:**
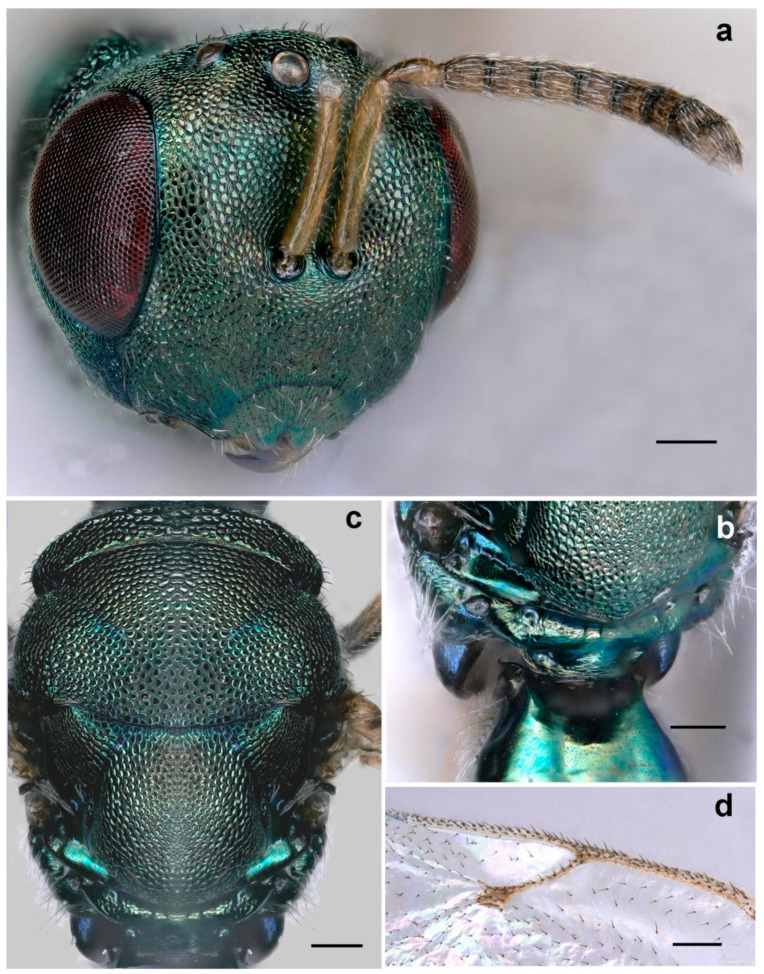
*Pteromalus* sp., female: (**a**) head and antenna, laterofrontal view; (**b**) mesoscutellum and propodeum, dorsal view; (**c**) mesosoma, dorsal view; (**d**) venation (scale bar, 100 µm).

*Pteromalus* sp. (Hymenoptera: Pteromalidae) (Figure 10 and Figure 11).

**Material examined:** 2♀♀, Iran: South Khorasan province, Birjand (32°51′59″ N, 59°13′55″ E), 1465 m, vii. 2017, A. Amini, leg.

**Diagnosis**. Some of the noticeable morphological characters of this undetermined species are POL about 1.6 times as long as OOL; clypeus bilobed, strigose dorsally (Figure 11a); antennal toruli located in the center of head (Figure 11a); antennal formula 1,1,2,6,3, all funiculars slightly longer than wide (Figure 10a); pronotum anteriorly carinate (Figure 11c); fore wing basally bare, with large open speculum, marginal vein as long as postmarginal vein, 1.5 times as long as stigmal vein (Figure 10b); propodeum mainly smooth, slightly reticulate anteriorly, without median carina (Figure 11b).

**Remark**. This species is a larval ectoparasitoid of BFF. Several species of the genus *Pteromalus* have been previously reared from fruit flies [54].

*Smicromyrme* (*Eremotilla*) *tekensis* Skorikov, 1935 (Hymenoptera: Mutilidae)

**Material examined**. 1♀, Iran: South Khorasan province, Mood (32°42′ N, 59°31′ E), 1839 m, Sarbishe, 33 km SE Birjand, vii.2013, A. Amini leg. 1♀, Birjand, Razg village (32°53′ N, 59°13′ E), 1470 m, i.2012, A. Amini, leg.

**Diagnosis**. The alate male of this species exhibits the following distinctive characters: mandible bidentate with well-developed subventral tooth, separated by excision; clypeus somehow flattened, without preapical teeth; head behind the eyes strongly convergent in dorsal view, without posterolateral angles. Apterous females have a triangular pygidial area, conspicuously widened basally with oblique striae throughout its length, lateral carina not widened apically; mesoscutellar scale well developed.

**Remark**. This species was reported from China, Kazakhstan, Mongolia, Tajikistan, Turkmenistan, Uzbekistan [55] and Iran [30]. It is a pupal parasitoid of BFF in soil.

*Smicromyrme* (*Astomyrme*) *nikolskajae* Lelej, 1985 (Hymenoptera: Mutilidae)

**Material examined**. 1♀, Iran: South Khorasan province, Birjand, Razg village (32°53′ N, 59°13′ E), 1470 m, 14.x.2012, A. Amini, leg. 1♀, 2♂, Same locality, iix.2013. 1♂, 33 Km SE Birjand, Sarbishe, Mood (32°42′ N, 59°31′ E), 1839 m, A. Amini, leg.

**Diagnosis**. The species is recognized by the following characters: mandible without subventral tooth; if an inconspicuous tooth is present, then the mandible has no excision; clypeus weakly elevated medially with two apical tubercles; antenna brownish-red, paler ventrally. In females, striae of the pygidial area not reaching the posterior margin, posterior third smooth; mesosoma with well-developed antero- and posterodorsal angles, dorsally more or less flattened (in lateral view); baso-medial pale spot of second metasomal tergum, 2.5–3.0 times distance between spot and medial apex of apical pale band of same tergite.

In the subgenus *Astomyrme,* this species is closely related to *Smicromyrme ausonius* Invrea, 1950, but the male of *S. nikolskajae* can be separated by its smaller body size (body size is larger in *S. ausonius*, 6.0–10 mm), the clypeus weakly elevated medially bearing two apical tubercles (concave with three preapical tubercles in *S. ausonius*), and antenna brownish-red, paler beneath (antenna black, rarely reddish-brown beneath in *S. ausonius*).

The female of *S. nikolskajae* is mostly smaller (3.2–4.0 mm) than *S. ausonius* (3.0–6.0 mm); the distance between baso-medial pale spots and the apical pale band on Gt2 in *S. nikolskajae* is about two times as long as *S. ausonius;* in the *S. nikolskajae* apical, 1/3 of the pygidial area is smooth, while in the *S. ausonius* apical, 1/4–1/5 of the pygidial area is smooth.

**Remark**. This species is known to exist in Kyrgyzstan and Tajikistan [55] and has also been reported to be in Iran [30].

**Key to the hymenopterous parasitoids of BFF, Carpomya vesuviana in Iran**

- Fore wing usually with enclosed cell, male apterous (only in Mutillidae)        **2**
  - Fore wing without enclosed cell (Figure 4j, Figure 6d, Figure 9c and Figure 10b)       **4**
- Gt1 node-like; pronotum usually indistinctly fused with mesothorax, pronotum medially less than one-third dorsal length of mesonotum-propodeum; Gs1 separated from Gs2 by a deep constriction; body covered with long hairs    **Mutillidae 3**
  - Metasoma without node-like segments; pronotum distinctly separated from and larger than mesonotum; Gs1 and Gs2 not separated by a constriction; body without long hairs    **Braconidae, *Fopius carpomyiae***
- In males, mandible beneath without subventral tooth, if with inconspicuous one then without excision; clypeus medially weakly elevated with two apical tubercles; in females, pygidial area with striae not reaching posterior margin, apically smooth; apical 1/3 of pygidial area not sculptured, smooth    ***Smicromyrme nikolskajae***
  - In males, mandible bidentate, beneath with well-developed subventral tooth separated by excision; clypeus flattened, without two preapical teeth; in females, pygidial area triangle, conspicuously widened basally with striae throughout; pygidial area with oblique striae    ***Smicromyrme tekensis***
- Pronotum more or less U-shaped in dorsal view; medial length usually less than one-quarter as long as mesoscutum; Gt1 several times as long as any other tergite and much wider than petiole, petiole cylindrical    **Diapriidae, *Coptera* nr. *silvestrii***
  - Pronotum rectangular, longer than mesoscutum medially; Gt1 shorter than other tergites and wider; petiole conical    **5**
- Pronotum short and transverse (Figure 10a); body finely sculptured, with green metallic reflection (Figure 10a,b and Figure 11a)            **Pteromalidae 6**
  - Pronotum long and shoulder-like, body coarsely sculptured (Figure 7b); body black without metallic reflection (Figure 5a,b and Figure 7a,b)    **Eurytomidae 7**
- Fu1 distinctly longer than pedicel; clypeus ventrally emarginated; propodeum smooth, with a median carina    ***Cyrtoptyx lichtensteini***
  - Fu1 shorter than pedicel (Figure 11a); clypeus ventrally bilobed (Figure 10a); propodeum smooth, without median carina (Figure 11b)    ***Pteromalus* sp.**
- Mesopleuron with horizontal ventral shelf in front of mesocoxal foramen, adscrobal carina of mesopleuron elbowed (Figure 4g); sublateral prepectus with a deep pit, its bottom not or rarely visible; epicnemium always completely delimited by a carina    ***Aximopsis augasmae***
  - Mesopleuron without ventral shelf, adscrobal carina of mesopleuron not elbowed, forming a single tooth in front of mesocoxa (Figure 8a); sublateral prepectus sometimes different; epicnemium laterally delimited by epicnemial carina, not ventrally    **7**
- Lower face with strigose supraclypeal area (Figure 6g); pedicel brownish-dark (Figure 6a); all coxae black, all femora medially black (Figure 5a)    ***Eurytoma pineticola***
  - Lower face often with punctured supraclypeal area (Figure 6c); pedicel yellow (Figure 7b); fore and mid coxae yellow, all femora mainly yellow (Figure 7a)    ***Eurytoma serratulae***

## 4. Discussion

Chemical insecticides are presently employed as major tools against BFF because the availability of potential biocontrol agents is very limited or they are unavailable [22]. In this study, we listed nine species of parasitoid wasps belonging to five families associated with BFF and provided new biological information. Some of these species can be useful potential control agents for this pest.

Amini et al. [30] reported *Cyrtoptyx lichtensteini* as a larval ectoparasitoid and *Galesus* nr. *silvestrii* as a pupal endoparasitoid of BFF. They also reared two mutillid species as pupal parasitoids of BFF in Eastern Iran [30]. Our study provides new information about the parasitoids of BFF and increases the number of parasitoids associated with this economically important pest to ten species. New findings of *Eurytoma pineticola* and *E. serratulae* and host–parasitoid association of all species were demonstrated by rearing for the first time. Among these parasitoids, *E. pineticola* and *C. lichtensteini* are the most abundant parasitoids on BFF and, after bioecological validation, these species could be recommended for biological control programs. While five braconid species and *Omphale* sp. (Eulophidae) were reported as parasitoids of the ber fly in India [27]. The egg–larval braconid *Fopius carpomyiae* is the first reported parasitoid of BFF in Iran. We did not rear it from BFF associated with *Z. jujuba,* but the species is known to parasitize this tephritid species on *Ziziphus spina-christi,* an indigenous species in Eastern Iran.

Eight species of parasitic wasps of the BFF from four families are now listed from Khorasan Iran. Most of these species are mainly active during the fruiting season in June–July when the maximum activity of the pests can be observed. This synchronization can be a positive agent in the future biological control program of the pest.

The parasitism rate of the studied parasitoids reached a range of 0.1% to a maximum of 1.6% (Figure 2) and 5.5% in total, which may be influenced by numerous biotic and abiotic factors such as host plant variety and climatic factors, respectively. The introduction of *F. carpomyiae* as an effective parasitoid in India and use in a classical biological control program can be proposed in the IPM program of BFF as an alternative for chemical control.

Within the listed parasitic wasps, *F. carpomyiae*, *E. pineticola* and *C. lichtensteini* can be proposed as candidates for future control programs. However, each of these species needs bioecological evaluation before mass production application. In contrast, the mutilid species have the minimum chance for biological control application against BFF, due to the difficulty of mass production and their extremely painful stings.

This result suggests that *Fopius carpomyiae*, which was introduced from the Oriental region with imported fruits, has not yet reached Eastern Iran. Bushehr in the south of Iran has a hot and humid climate with a mean temperature of 25 °C (up to 50 °C in summer) and a total annual rainfall of 170 mm, which is similar to the Indian climate, the origin country of *F. carpomyiae*. South Khorasan in eastern Iran has a desert and dry climate with a mean annual temperature of 33 °C in summer and 14 °C in winter, with a total annual rainfall of 134 mm, which seems inappropriate for this parasitoid. The elevation of Bushehr is 18 m while it is between 600 to 3600 m in South Khorasan. These abiotic could be the possible cause of the absence of *F. carpomyiae*. However, we envisage its presence in the near future. Although the parasitism rate of obtained parasitoids is low, it can be evaluated on a larger scale to have an accurate evaluation. Additionally, including *F. carpomyiae* as an important species on this list can increase the parasitism of BFF. Its parasitization rate was estimated to be 21% to 26.7% in the south of Iran [17]. Jena et al. [22] believe its distinct ovipositor is very suitable to parasitize the hidden eggs of BFF in fruits. On the other hand, importing populations of this exotic parasitoid to pest-infested areas in eastern Iran can be useful as a practical proposal to reduce pest damage.

Most of the obtained parasitoids are mainly active during June–July with an average temperature of 31 °C and low rainfall when the maximum activity of the pests can be observed; this synchronization can be a positive factor in the future biological control program of the pest. The successful management of BFF with parasitoids can be made through the augmentative release that requires developing a mass production program and efficacy testing techniques.

## Figures and Tables

**Table 1:** Hymenopterous parasitoids of BFF, *Carpomya vesuviana* (Diptera: Tephritidae) infesting jujube fruit, *Ziziphus jujuba* in eastern Iran and their mode of parasitism and activity periods.

Family	Species	Mode of Parasitism	Activity Period
Braconidae	*Fopius carpomyiae* (Silvestri, 1916)	egg-larval endoparasitoid	-
Diapriidae	*Coptera* nr. *silvestrii* (Kieffer, 1913)	Solitary puparia endoparasitoid	July
Eurytomidae	*Aximopsis augasmae* (Zerova, 1977)	Solitary larval ectoparasitoid	July
	*Eurytoma pineticola* Zerova, 1981 *	Solitary larval ectoparasitoid	June
	*Eurytoma serratulae* (Fabricius, 1798) *	Solitary larvae	July
Pteromalidae	*Cyrtoptyx lichtensteini* (Masi, 1921)	Larval ecoparasitoid	July
	*Pteromalus* sp.	larvae	July
Mutillidae	*Smicromyrme* (*Astomyrme*) *nikolskajae* Lelej, 1985	Solitary ectoparasitoid of puparia	October
	*S.* (*Eremotilla*) *tekensis* Skorikov, 1935	Solitary ectoparasitoid of puparia	January & July

* new record.

## Data Availability

The data presented in this study are available on request from the corresponding author.

## Abbreviations

**C1–3**: first to third clavomeres; **Fu1, Fu2, etc.**: first funicular, second funicular, etc.; Gt1-n: gastral terga 1-n.; **Gs1-n**: gastral sternite 1-n; **OOL**: Oculo-ocellar line; **POL**: Posterior-ocellar line; **HMIM**: Hayk Mirzayans Insect Museum, Iranian Research Institute of Plant Protection, Tehran, Iran; **NHMUK**: Natural History Museum, London, U.K.
